# Is neonatal phototherapy associated with a greater risk of childhood cancers?

**DOI:** 10.1186/s12887-022-03412-0

**Published:** 2022-06-22

**Authors:** Fatemeh Sabzevari, Reza Sinaei, Bahareh Bahmanbijari, Simin Dehghan Krooki, Azam Dehghani

**Affiliations:** 1 Department of Pediatrics, School of Medicine, Afzalipour Hospital, Kerman University of Medical Sciences, Kerman, Iran; 2 Department of Pediatrics, School of medicine, Kerman University of Medical Sciences, Kerman, Iran; 3 Endocrinology and Metabolism Research Center, Institute of Basic and Clinical Physiology Sciences, Kerman University of Medical Sciences, Kerman, Iran; 4 Department of Pediatrics, School of Medicine, Endocrinology and Metabolism Research Center, Institute of Basic and Clinical Physiology Sciences, Kerman University of Medical Sciences, Kerman, Iran; 5 Clinical Research Development Unit, Afzalipour Hospital, Kerman University of Medical Sciences, Kerman, Iran; 6 Gastroenterology and Hepatology Research Center, Institute of Basic and Clinical Physiology Sciences, Kerman University of Medical Sciences, Kerman, Iran

**Keywords:** Childhood, Cancer, Malignancy, Neonatal phototherapy, NNPT, Hyperbilirubinemia

## Abstract

**Background:**

Neonatal phototherapy (NNPT) has long been used as an effective and relatively safe method of treating neonatal hyperbilirubinemia. Considering the subsequent evidence of long-term impacts of NNPT such as malignancies, this study was conducted to evaluate the relationship between NNPT and childhood cancers.

**Methods:**

This case-control study assessed 116 children up to 4 years old with every kind of cancer referred to the Oncology department of Afzalipour hospital, Kerman, Iran, from 2011 to 18. Moreover, 116 pediatric patients without cancer hospitalized at the same Center were included after sex and age matching as the control group. The history of phototherapy and its duration were evaluated in these two groups.

**Results:**

We found no association between the NNPT and malignancies in children. However, high intensive phototherapy was higher historically among affected cancerous patients than in non-cancerous cases without any statistically significant difference (25% vs 19%; *P* = 0.26). Maternal educational level and history of maternal infection during pregnancy, which initially appeared to be two factors associated with malignancy in single variable regression analyses, were not significant based on the adjusted models.

**Conclusions:**

The results did not show a positive correlation between NNPT and childhood cancers, which may partly be due to the relatively small sample size of the study. However, some other evidence is worrisome enough that NNPT should not be considered risk-free. Additional multi-centric studies should be undertaken to specify that phototherapy is really safe.

**Supplementary Information:**

The online version contains supplementary material available at 10.1186/s12887-022-03412-0.

## Introduction

A number of characteristics of childhood cancers provide clues to the timing of culprit factors. Embryonal cell neoplasia (such as neuroblastoma and retinoblastoma) may be originated in early infancy even shortly after birth. The peak of childhood acute lymphoblastic leukemia (ALL) during the third and fourth years of life suggests a possibility of a discrete leukemogenic event, which may occur during the perinatal period [1]. Development of ALL in pediatrics is believed to involve at least two genetic “hits”; the first “hit” is supposed to be often accomplished in the prenatal period [2]. Additionally, some other factors such as birth order, birth weight, sex, and oxidative stress such as neonatal oxygen supplementation have been suggested as additional risk factors [3, 4].

Bilirubin as an endogenous antioxidant in the neonatal period is released as part of the normal life cycle breakdown of the erythrocytes [5]. At high concentration levels, bilirubin turns the skin yellow and can cause a devastating brain impact, known as kernicterus [6]. Given the permanent neurodevelopmental handicaps of hyperbilirubinemia, in 1985, the National Institute of Child Health and Human Development reported that NNPT was as effective as exchange transfusion in preventing neurological squeals [7]. Since then, NNPT (especially with blue light) has been widely exploited as the initial therapy of choice in clinical practice [8, 9]. The NNPT acts not only in the skin but also in capillary circulation under the skin, reducing the serum bilirubin concentrations by converting bilirubin through structural photo-isomerization and photo-oxidation into excretable products [10]. Phototherapy at the 425–475 nm wavelength with a peak of 450–460 nm is an impressible method for the treatment of neonatal hyperbilirubinemia [11]. Unfortunately, a tiny portion of ultraviolet (UV) light accounts for a minor proportion of the traditional blue-green phototherapy that is gradually linked with the production of inflammatory factors in the skin, as well as gene expression [12, 13].

A possible relationship between NNPT and several possibilities especially long-term cancers has been raised in recent years. Some experts believe that hyperbilirubinemia in “healthy” neonates without other complications is still being over treated [14].

A variety of studies have shown a correlation between hyperbilirubinemia and Deoxyribonucleic acid (DNA) damage in hyperbilirubinemic newborns [15, 16]. The negative impacts of phototherapy have previously been investigated on the antioxidant defense system of jaundiced neonates [17]. Mesbah-Namin et al. revealed that phototherapy during hyperbilirubinemia is able not only to induce apoptosis in lymphocytes but also to affect indirectly DNA integrity. The double-strand breaking (DSB) levels were substantially much higher in the phototherapy-treated group than in controls before incubation but decreased remarkably after the incubation period. No statistical differences were found between the two control groups before and after the incubations. The frequency of apoptotic cells showed no substantial differences among the three groups before incubation; however, it substantially increased in the phototherapy-treated group after the incubation [18]. In 1979, Speck WT et al. investigated the mutagenicity of the blue light in the laboratory environment. They noted that phototherapy had a role in oxidative stress, DNA damage, and consequently in the pathology of cancer. They recommended that the advantages of phototherapy should be considered concerning its potential hits, especially in prophylactic administrations [19]. Whether all of these situations are expressed in the pathogenesis of malignancies is a matter of concern.

In addition to the obvious effect of phototherapy in reducing the neonatal serum bilirubin levels, this therapeutic modality promotes the serum tumor necrosis factor-α (TNF-α) level that can provoke the proliferation of some tumor cell lines [20, 21].

Nevertheless, it is not yet fully known whether NNPT will induce long-term impacts since only a few clinical studies have been performed. Herein, we aimed to evaluate the relationship between neonatal phototherapy and childhood cancer.

## Materials and methods

### Ethics approval

This research, involving human participants, was performed in accordance with the Declaration of Helsinki and approved by the Ethics Committee of Kerman University of Medical Sciences, Kerman, Iran (Code: IR.KMU.AH.REC.1398.056).

### Study design

This case-control study assessed 232 children up to 4 years old including 116 cancer patients and 116 controls. 116 out of 232 participants were cases with each kind of malignancies referred to the Oncology department of Afzalipour hospital of Kerman, southeastern Iran, between 2011 and 2018. All childhood hematological and solid tumors were included in the study. Control group were 116 children hospitalized for other reasons (except malignancies) in pediatric wards after sex and age matching.

The main variable in this study was the history of receiving intensive phototherapy in the neonate, which was recorded in the participants’ files. Other variables including birth weight, maternal age, maternal education level, and carcinogenic factors including a family history of cancer and maternal or paternal smoking were enrolled.

### Inclusion and exclusion criteria

Inclusion criteria: In the case group, all children under four years old hospitalized in the referral oncology department of Afzalipour hospital were enrolled in the study. Children under four years old admitted to pediatric wards while not having clues in favor of cancers in history, physical examination, and laboratory investigations were included after sex and age matching as the control group.

The exclusion criteria were considered as below:

1. Children over 4 years old
2. The presence of syndromes related to childhood malignancies including Down, Von Hippel-Lindau, Beckwith-Wiedemann, and Neurofibromatosis syndromes
3. Congenital major anomalies
4. History of prematurity (gestational age below 35 weeks)
5. Lack of permission by the children’s parents
6. Incomplete or doubtful data
7. Children with a history of blood exchange during their neonatal period (tend to require a shorter period of phototherapy)

### Data collection and statistical analysis

Demographic characteristics such as age, gender, gestational age, birth weight, maternal age, maternal educational state, and carcinogenic factors including a family history of cancer, maternal or paternal smoking, phototherapy, and its duration were collected. All data were collected via pre-prepared checklists and analyzed in version 25 of SPSS software to describe both data using frequencies as both qualitative and quantitative variables. In addition, the univariate logistic regression and Mantel-Haenszel test for controlling Neonatal weight were used if needed to analyze the data. Considering a 95% confidence interval (CI) and test power of 80%, a *p*-value less than 0.05 was considered statistically significant.

## Results

A total of 232 children under 4 years old including 116 cancer patients (case group) and 116 children without cancer (control group) were enrolled in this study.

The most common malignancies in the cancer group were acute lymphoblastic leukemia (ALL) and acute myeloid leukemia (AML) observed in 74 (63.8%) and 11.2% of children, respectively. Afterward, neuroblastoma, rhabdomyosarcoma, and osteosarcoma were respectively the most common tumors.

More than half the children in the study were boys (*n* = 144, 62.1%). The Demographic data of both case and control groups of the study are presented in Table 1.

**Table 1:** Demographic data of case (cancer patients) and control (Non-cancer patients) of the study

		Cancer patients		Non-cancer		Total	
		n	%	n	%	n	%
**Age**	**1–2-y**	24	20.7	24	20.7	48	20.7
	**2–3-y**	37	31.9	37	31.9	74	31.9
	**3–4-y**	55	47.4	55	47.4	110	47.4
**sex**	**Male**	72	62.1	72	62.1	144	62.1
	**female**	44	37.9	44	37.9	88	37.9
**Neonatal weight**	**< 2500 g**	21	18.3	21	18.9	42	18.6
	**2500–3999 g**	88	76.5	84	75.7	172	76.1
	**> 4000 g**	6	5.2	6	5.4	12	5.3
**Mother age**	**< 20-y**	5	4.3	5	4.3	10	4.3
	**20–24-y**	31	26.7	19	16.5	50	21.6
	**25–29-y**	34	29.3	36	31.3	70	30.3
	**30–34-y**	22	19	30	26.1	52	22.5
	**< 35-y**	19	20.7	25	21.7	49	21.2
**Mother educational level**	**Illiterate**	11	9.6	2	1.7	13	5.6
	**Elementary**	16	13.9	11	9.5	27	11.7
	**High school**	28	24.3	36	31	64	27.7
	**Higher education**	60	52.2	67	57.8	127	55
**History of phototherapy**	**No**	87	75	94	81	181	78
	**Yes**	29	25	22	19	51	22
**Urban and rural life**	**Rural**	23	20.4	21	18.1	44	19.2
	**Urban**	90	79.6	95	81.9	185	80.8
**History of medicine**	**No**	104	90.4	108	93.1	212	91.8
	**Yes**	11	9.6	8	6.9	19	8.2
**History of radiography**	**No**	104	91.2	113	97.4	217	94.3
	**Yes**	10	8.8	3	2.6	13	5.7
**Breast feeding**	**No**	11	9.6	11	9.5	22	9.6
	**Yes**	77	67.5	71	61.2	148	64.3
	**Both (+ formula)**	26	22.8	34	29.3	60	26.1
**History of maternal addiction**	**No**	110	95.7	113	97.4	223	96.5
	**Yes**	5	4.3	3	2.6	8	3.5
**History of maternal infection**	**No**	93	80.9	110	94.8	203	87.9
	**Yes**	22	19.1	6	5.2	28	12.1

The frequency of sex and age distribution was the same in both groups. The patients aged 3–4 years and 2–3 years had the highest frequency with 47.4 and 31.9% of total participants, respectively. The birth weight was between 2500 and 3999 g in 172 participants. Only 42 children (18.1%) weighed less than 2500 g at birth. However, 76.5 and 75.7% of participants in the case and control groups were in the 2500–3999 g weight group, respectively.

148 out of 312 participants (63.8%) had a history of breastfeeding, while 60 others (25.9%) had a history of breastfeeding and formula together usage. The maternal age at the birth date of under 20 years, 20–24, 25–29, 30–34, and over 35 years were observed in 10 (4.3%), 50 (21.6%), 70 (30.1%), 52 (22.4%), and 50 (21.6%) of the mothers, respectively. While 13 mothers (5.6%) were illiterate, 127 others (54.7%) had a bachelor’s or higher degree. Only one mother and one father had a history of cancer compatible with a brain tumor and chronic myelobelastic leukemia (CML), respectively. No history of cancer was found in any of the siblings. 185 participants (79.7%) had urban life and the rest lived in rural areas. 19 mothers (8.2%) had a history of drug intake including anti-epileptic and insulin during pregnancy. Eight mothers (3.4%) had a history of opioid and heroin abuse. The history of maternal infectious disease during the pregnancy was observed in 28 cases, including the common cold symptoms in eight, urinary tract infection in seven, influenza in seven, malaria in two, and pneumonia in one participant.

The relationship between the variables in the two groups is presented in Table 2, where based on multivariable analyzes, none of the variables were significant between the groups. Both Crude and adjusted odds ratios for estimating risk and controlling for confounding bias were evaluated in this study.

**Table 2:** Determining the relationship between variables in case and control groups

	crude		Adjusted	
	OR (CI 95%)	P. Value	OR (CI 95%)*	P. Value
**Age**	1 (0.79–1.39)	1		
**Sex**	1 (0.58–1.7)	1		
**Neonatal weight**	0.97 (0.56–1.7)	0.94		
**< 2500 g**	0.85 (0.47–1.09)	0.81		
**2500–3999 g**	0.99 (0.55–1.7)	0.94		
**> 4000 g**	0.76 (0.13–1.22)	0.43		
**Mother age**	1.15 (0.92–1.44)	0.20	0.10 (0.01–1.12)	0.062
**Mother educational level**	1.39 (1.03–1.88)	0.03	3.9 (0.11–134.09)	0.45
**History of phototherapy**	0.70 (0.37–1.31)	0.268	1.38 (0.03–55.53)	0.864
**History of radiography taking**	0.27 (0.07–1.03)	0.056	0	0.99
**History of maternal infection**	0.23 (0.09–0.59)	0.002	1.22 (0.29–5.1)	0.77
**History of medicine**	0.7 (0.27–1.81)	0.46		
**Maternal addiction**	0.58 (0.13–2.50)	0.46		
**Neonatal feeding type**	1.22 (0.77–1.92)	0.37		
**Urban and rural life**	1.15 (0.59–2.23)	0.66		

*based on logistic regression that were adjusted by mother age, mother educational level, history of radiography taking, history of maternal infection

The odds ratio (OR) was not significant for children with a history of phototherapy compared to those without a history of phototherapy (OR: 0.7 CI95% (0.37–1.31)). Moreover, age, gender, maternal age, maternal addiction, neonatal nutrition, and urban or rural life were not significant, but the history of maternal infection and maternal educational level were significant.

According to the modified logistic regression test that was modified based on the variables of maternal age, maternal educational level, history of NNPT, and maternal infection, the OR between groups, with and without a history of receiving NNPT, was again insignificant (OR: 1.38 CI95% (0.03–55.53)).7.

## Discussion

The results showed that the history of NNPT, regardless of other factors and variables, had no significant relationship with childhood cancer. These impacts along with some other factors, including maternal educational state and maternal infections during pregnancy are of great importance.

According to a systematic review conducted on the incidence of childhood cancer in Iran, the incidence of childhood cancer among Iranian boys and girls up to 14 years was higher than in other countries. The incidence was higher in boys aged up to 4 years old, and the lowest incidence rate of cancer among boys and girls occurred respectively in age groups of 10–14 years and 5–9 years [22]. However, we chose the age group under four years to minimize other possible confounding factors that may occur over time.

The higher incidence of childhood cancer in Iran might be partly due to the introduction of antenatal and perinatal factors such as imaging, NNPT, and other possibilities. Childhood cancers are representing partly different in geographical locations, worldwide. However, in Kerman as a referral center for oncologic problems in the southeast of Iran, the most common malignancies in the cancer group were ALL and AML, which occurred together in about 75% of children, followed by neuroblastoma, rhabdomyosarcoma, and osteosarcoma. Whether childhood cancer is more common in Iran than the average of the world, and which factors play a role in its occurrence has occupied the minds of researchers in recent years.

Most previous studies have investigated phototherapy as a risk factor for childhood cancers including hemategenous malignancies and solid tumors. We included some relevant articles investigating the impact of neonatal phototherapy on childhood cancers in the Table S1 (appendix).

In a retrospective large cohort study” first published online in June 2010, Brewster DH et al. evaluated the risk of developing skin cancers in young adults following the exposure to blue-light NNPT [23]. There are several limitations in Brewster’s study including no information regarding blue-light exposure, limited statistical power, and the absence of real data regarding sun exposure and several other confounding factors during childhood. Similarly, in the study conducted by Wiecker et al., NNPT was not associated with an increased risk of developing melanocytic cancers [24]. In contrast, Matichard et al. showed that intensive NNPT increased the melanocytic nevus count during childhood [25].

The California Late Impact of Phototherapy Study (CLIPS) analyzed data from 5,144,849 infants born at ≥35 weeks of gestation in California hospitals in the ten years between 1998 and 2007. The study used administrative data that linked the billing code of phototherapy and the diagnosis code for pediatric cancers. The absolute numbers affected in this study out of the 5 million infants were only 58 cases who received phototherapy and later developed cancer. The strongest association in this study was a 1.6-fold increased risk of AML, following the risk of Wilms tumor that also rose to statistical significance in this study [26].

The other large cohort, the Late Impact of Getting Hyperbilirubinemia of photoTherapy (LIGHT) study, analyzed data from nearly 500,000 children born at ≥35 weeks of gestation in the Kaiser Permanente Northern California healthcare system in the twelve years between 1995 and 2011. Although the associations between phototherapy and childhood cancers were not statistically significant, an association with liver cancers and leukemia, particularly non-lymphocytic forms, was observed [27]. In a retrospective cohort of 786,998 infants born in hospitals in Quebec, Canada, between 2006 and 2016, phototherapy was associated with more than two times the risk of late-onset solid tumors, including brain and central nervous system (CNS) related cancers compared to unexposed children (HR 2.26, 95% CI 1.34–3.81) [28].

In another study conducted by Kadivar and colleagues, 500 children up to 14 years with every kind of cancer and 500 children without cancer were enrolled in the study. NNPT was not correlated with childhood cancer; however, it increased the risk of cancer by 55–88% when it is accompanied by the male gender, maternal age > 35 years during pregnancy, and smoking by the father [29]. Similar results were found in a retrospective cohort of 342,172 infants born ≥32 weeks with 9.5 years follow up, while 18,797 of whom were exposed to phototherapy, with a statistically significant increased risk of childhood malignancies and benign tumors [30].

In contrast, in another study on 55,120 neonates treated with phototherapy conducted between 1977 and 1989, no significant risk was found regarding the relationship between NNPT and childhood leukemia [31]. Similar results were found in two other studies conducted by Seppälä LK et al. and Dixon et al. [32, 33].

In our study, the risk of childhood cancer was not statistically significant. We are experiencing a relatively different geographical pattern of malignancies in this region. We did not have melanocytic cancers, and CNS malignancies are to some extent less frequent in Kerman province than the world’s average. Most previous studies investigated phototherapy as a risk factor for childhood cancers including hemategenous malignancies [26–30, 34] and solid tumors [26–29, 34]. Nevertheless, some studies have results consistent with ours, highlighting the NNPT as a relatively risk-free therapeutic method [31–33]. Finally, in a systematic review and meta-analysis of 11 included articles, phototherapy was significantly associated with an increased risk of all types of cancer, especially leukemia (RR = 1.28; 95% CI, 1.08–1.51) [34]. However, our study had some limitations. Considering the cross-sectional nature of the study, the lack of sufficient data of participants led to the initial exclusion of a large number of samples. Furthermore, the presence of several factors such as agricultural pesticides that may cause cancers led us to consider the age of participants slightly less than other studies. Nevertheless, despite not having a large sample size, both Crude and adjusted odds ratios for estimating risk and controlling for confounding bias were evaluated in this study. According to the modified logistic regression test that was modified based on the variables of maternal age, maternal educational level, history of NNPT, and maternal infection, the OR between groups, with and without the history of receiving phototherapy, was again insignificant. Although this study did not find a relationship between birth weight and childhood cancers, unlike many studies [34], using the Mantel-Haenszel test, we considered the confounding effect of birth weight, which has not shown a significant relationship between NNPT and childhood cancer.

## Conclusion

The brain damage and hearing loss from high bilirubin levels in the absence of effective and less dangerous alternative therapeutic procedures have made phototherapy a passable and relatively safe procedure.

Evidence raise enough doubts regarding a possible link between childhood onset cancers and neonatal light therapies. However, the power of detecting the association between neonatal phototherapy and childhood cancer is limited due to several factors. Since our sample size was relatively small, we are concerned enough that NNPT should not be considered a risk-free therapeutic strategy. Considering these limitations, adjusted odds ratios for controlling the confounding bias were evaluated in this study.

Nevertheless, due to the small increased risk of childhood cancers in several studies such as CLIPS, making the phototherapy decisions should be preserved for infants with high bilirubin levels compatible with current treatment guidelines. Certainly, large multi-centric studies will have more reliable results.

## Supplementary Information

**Additional file 1.**

## Data Availability

All data generated or analyzed during this study are included in this published article.
