# Supplementary material for: Is neonatal phototherapy associated with a greater risk of childhood cancers?
Source: BMC Pediatr. 2022 Jun 22;22:356. doi: 10.1186/s12887-022-03412-0 (PMC9215034; doi:10.1186/s12887-022-03412-0)
Supplement: Supplementary file 1 — Additional file 1. [file 12887_2022_3412_MOESM1_ESM.docx]

Table S1. The impact of neonatal phototherapy on childhood malignancies based on previous studies

| Authors | Subjects/Study | Main findings | Cancer type | The risk of cancer by phototherapy^1^ |
| --- | --- | --- | --- | --- |
| Matichard E, et al^25^ | Case-control prospective study of 58 children aged 8-9-y including 18 cases with history of phototherapy | Multi-variate analysis showed a statistically significant correlation with nevus count and sizes more than 2 mm. | Melanocytic Nevus | A |
| Wickremasinghe AC, et al^26^ | 5144849 infants born at≥ 35 weeks gestation from 1998-2007. | Cancer was diagnosed in 58/178017 infant with history of phototherapy and 1042/4966832 infants without such therapy (RR 1.6; 95% CI, 1.2-2.0, p=0.002) | Myeloid leukemia, Kidney cancer | B |
| Newman TB, et al^27^ | A retrospective cohort study of 499621 neonates born at≥ 35 weeks gestation during 1995-2011. | Higher incidence of cancer in whom received phototherapy (p=0.01). | Non-lymphocytic leukemia and liver cancer | A |
| Auger N, et al^28^ | A retrospective cohort of 786998 infants born between 2006-2016 | The exposure groups were phototherapy group (32314) and untreated jaundice (91855). The incidence of childhood cancer was higher in cases with phototherapy and untreated jaundice compared to unexposed infants. | Phototherapy appeared to be associate with late onset solid tumors, including brain/central nervous system cancers. A similar trend was however less apparent for hematopoietic cancer. | B. |
| Kadivar M, et al^29^ | 500 children up to 14-y with every kind of cancer and 500 children without cancer were enrolled. | Neonatal phototherapy was not correlated with childhood cancer. But, it will increase the risk of cancer by 55%-88% when it is accompanied by the male gender, maternal age> 35-y during pregnancy, and smoking by father. | ALL, Neuroblastoma  Slightly increase when it is accompanied by the male gender, maternal age> 35-y (by 55%), and smoking by father (by 88%). | B |
| Bugaiski-Shaked A, et al^30^ | A retrospective cohort of 342172 infants who born≥ 32 weeks gestation, of them 18797 were exposed to phototherapy. Median follow up time was 9.5-y | Phototherapy was associated with a significant increased risk of childhood malignancies and benign tumors. | All hematopoietic cancers and benign tumors especially leukemia, but not solid tumors and lymphoma | A |
| Hemati Z, et al^34^ | Meta-analysis of 11 articles | Phototherapy was significantly associated with an increased risk of all types of cancer (RR = 1.28; 95% CI, 1.08-1.51). | All types of cancer (RR = 1.28; 95% CI, 1.08-1.51), especially leukemia | A |
| Brewster DH, et al^23^ | A retrospective cohort of 77518 participants, while 5868 received NNPT. | No significant evidence of an excess risk of skin cancers (RR = 1.40, 95% CI, 0.17-5.04; p=0.834). Two cases of melanoma occurred. | Melanoma | C |
| Wiecker TS, et al^24^ | A cross-sectional study in 1812 children aged 2-7-y, while phototherapy with blue lamps was performed in 333 children. | Phototherapy was not a significant factor to develop melanocytic nevi (p=0.21). | Melanocytic Nevus | C |
| Olsen JH, et al^31^ | Over 55120 neonates treated by phototherapy were identified between 1977- 1989. | The incidence of leukemia in 34 children was not significant. | Leukemia | C |
| Seppälä LK, et al^32^ | A case-control study on 2029 patients and 10103 matched controls | Preterm birth and mechanical ventilation were associated with an increased risk of childhood cancer. Phototherapy was not associated with an increased risk of childhood cancer. | AML, Germ cell tumors, and Retinoblastoma | C |
| Dixon F, et al^33^ | A study on 1072957 infants in Australia, between 2000 and 2011. | The only therapy used in the NICU that was independently associated with an increased risk of cancer was nitric oxide (Age of cancer diagnosed was four month-five years). | All malignancies, especially hepatic tumors. | C |

1- A: significant risk, B: Slightly increased risk, C: No significant risk
